# Efficacy and safety of chimeric antigen receptor T-cell (CAR-T) therapy in hematologic malignancies: a living systematic review (protocol)

**DOI:** 10.12688/openreseurope.14390.1

**Published:** 2022-03-15

**Authors:** Leire Leache, Marta Gutiérrez Valencia, Luis Carlos Saiz, Juan Erviti, Maria Ximena Rojas Reyes

**Affiliations:** 1 Unit of Innovation and Organization, Navarre Health Service, Pamplona, Tudela 20, 1st floor, 31003, Spain; 2 Institut d'Recerca-Servei d'Epidemiologia Clínica i Salut Pública, Hospital de la Santa Creu i Sant Pau, Barcelona, Carrer de Sant Quintí, 08041, Spain

**Keywords:** Living systematic review; Chimeric Antigen Receptor T- Cell therapy; living evidence synthesis; hematologic malignancies; CAR-T infusion to transplantation.

## Abstract

**Objective:** To determine the efficacy and safety of CAR-T therapy in the treatment of patients with hematologic malignancies, in comparison with other current therapies.

**Design**

A living systematic review.

**Methods**

Randomized trials evaluating the effect of CAR-T therapy versus other active treatments, hematopoietic stem cell transplantation, best supportive care or any other intervention in patients with hematologic malignancies will be considered for inclusion. If we find no direct evidence from randomized controlled trials, non-randomized primary studies will also be retrieved. Each of the identified studies will be independently assessed by two authors, both of whom will extract the information and evaluate the risk of bias. Efficacy measures include overall survival rate, overall response rate, complete response/remission (CR) rate, partial response/remission (PR) rate, relapse from CR, progression-free survival, and time interval between CAR-T administration and transplantation. Safety measures will include: serious adverse events, incidence of cytokine-release syndrome, graft-versus-host disease, neurotoxicity, and total adverse events. Quality of life will also be assessed. Meta-analyses will be carried out to summarize the results. Quality of the evidence will be determined using the GRADE approach. A living version of the review will be accessible in an open manner until there is solid evidence to respond to the review objective. Updated versions of the evidence synthesis will be published whenever substantial modifications make it necessary.

## Plain language summary

Research has been carried out over the last few years to assess the efficacy and usefulness of Chimeric Antigen Receptor T-Cell (CAR-T) therapy in hematologic malignancies. The present review aims to determine the efficacy and safety of CAR-T therapy in the management of patients suffering hematologic malignancies.

Randomized controlled trials (RCT) that answer our research question or non-randomized primary studies, in case we found no direct evidence of RCT, will be retrieved. The Epistemonikos database will be used for the identification of potentially eligible studies. We will identify studies meeting our inclusion criteria. We will evaluate The risk of bias and the quality of the evidence will be determined with specific tools. We will monitor the newly published evidence every two months, searching for relevant studies that could indicate changes in the available evidence. This monitoring process will last 12 months.

## Background

### Condition or domain being studied

Chimeric antigen receptors (CARs) show a high affinity to bind effector cells of the immune system, such as T cells ^1^. This cancer immunotherapy enables enhancement of the immunological response against malignant cells. Although the development of CAR T-cell (CAR-T) cell therapy started more than 20 years ago ^2^, the first steps in their transfer to clinical practice are now taking place, following the recent authorization of different CAR-T therapies by the U.S Food and Drug Administration and the European Medicines Agency.

### Why it is important to do this review

The earliest and most extensive research with CAR-T therapy has been carried out in hematologic malignancies, where it has pointed to high response rates in patients who generally have very poor prognosis such as refractory acute lymphoblastic lymphoma, diffuse large B-cell lymphoma, or multiple myeloma ^3– 5^. Few therapeutic options are available in this setting, and CAR-T therapy is showing encouraging results.

B-cell maturation antigen (BCMA) and CD19-targeting CAR-T cell therapies are the ones that are at a more advanced stage of research and have shown the best results so far ^6^. At this time, CAR-T cells directed to other targets for treating solid tumors and infectious or autoimmune diseases are emerging ^7, 8^.

Clinical studies have suggested that CAR-T cell therapies can be even curative in patients who are responsive. However response rates can vary in different pathologies or depending on the characteristics of the patient or the disease. There is also a possibility of resistance and relapses. Moreover, these therapies can associate important adverse events such as cytokine release syndrome, neurologic adverse events and B-cell aplasia, which may lead to serious consequences ^1^. To date, clinical trials have been mainly performed in terms of a single-arm design. Consequently, systematic reviews have focused on synthesizing overall response rates of CAR-T cell therapy, without providing results compared with a control group ^9– 12^. Direct comparisons between different CAR T-cells are also lacking.

The encouraging results obtained in non-comparative studies have allowed the approval of these therapies and their progressive introduction into clinical practice. However, all the above limitations do not make it possible to clarify in which patients there is a clearly favorable risk-benefit ratio or what place in therapy should be reserved to the existing therapies. Numerous phase III randomized clinical trials are already underway that will shed light on these questions in the near future. The development of CAR-T cell therapies will continue to expand and new CAR-T cells will emerge. Therefore, a living synthesis of the evidence with regular updates can be positioned as the best method to incorporate emerging evidence in a timely manner.

Considering the characteristics of the available evidence, the width variability of the SRs currently published, and taking into account that the evidence on this therapy is on the rise ( *i.e.* new studies have been published and other studies are going to be published in the near future), we propose to carry out a living systematic review that will make it possible to develop a rigorous and updated synthesis of the evidence on the efficacy and safety of CAR-T therapy in patients suffering from hematological malignancies. This SR will be developed as part of the *Living Evidence to Inform Health Decisions* project, which supports health system organizations in the implementation of a living process for the development of the synthesis of the evidence to inform health decisions ^13^.

### Objective

To determine the efficacy and safety of CAR-T therapy in the treatment of hematologic malignancies, in comparison with other current therapies

## Methods

A living systematic review. This protocol is reported in line with the Preferred Reporting Items for Systematic Reviews and Meta-Analyses Protocols (PRISMA-P). It shares a common methodological approach that has been defined on the basis of previous developments, as part of the Living Evidence to inform health decisions project ^13^ for developing multiple living systematic reviews and living overviews of reviews.

### Types of studies

Randomized controlled trials (RCTs) will be prioritized. In the absence of evidence from RCTs, non-randomized controlled studies (quasi-experimental studies, cohort studies, case-control studies) will be considered for inclusion. Studies with other designs, such as those with historical controls and single-arm studies will not be considered. Studies must analyze at least one primary or secondary outcome.

### Types of participants

Studies including participants diagnosed with hematologic diseases, such as multiple myeloma, leukemia and lymphoma of any type will be included. Both adults (≥ 18 years) and pediatric patients will be considered. Untreated patients, as well as patients previously treated, will be included, irrespective of the type of treatment or treatment line.

### Intervention

Any CAR-T therapy type will be considered regardless of the T-cell origin (allogenic or autologous), target antigen, costimulatory domain, or infusion dose.

### Comparator

The comparator will consist of chemotherapy or any other active pharmacologic treatment, hematopoietic stem cell transplantation (HSCT), best supportive care or any other intervention. 

### Outcome variables

***Efficacy assessment***

The primary outcome will be overall survival (OS).

Secondary efficacy variables will be mainly based according to the Center for International Blood & Marrow Transplant Research (CIBMTR) criteria ( https://www.cibmtr.org/manuals/fim/1/en/topic/none) and consist of:

Overall response rate (ORR) (proportion of patients who have a PR or CR)

- Complete Response/Remission (CR) rate (including Stringent Complete Response (sCR) and negative minimal residual disease (MRD) when provided)
- Partial Response/Remission (PR) rate (including Very Good Partial Response (VGPR) and positive MRD when provided)
- Relapse from CR
- Progression-free survival (PFS)
- Time period between CAR-T administration and transplantation

***Safety assessment***

Secondary safety outcomes will be the following:

- Serious adverse events (SAE)
- Incidence of cytokine-release syndrome (grade ≥3)
- Graft-versus-host disease
- Neurotoxicity (grade ≥3)
- Total adverse events (grade ≥3)
- Quality of life (QoL)

All efficacy and safety outcomes will be measured at the longest reported follow-up. For analyzing efficacy outcomes, variables will be considered as reported by the individual studies as long as internationally accepted criteria are used. The homogeneity of the criteria used for determining the outcomes in the different studies will be analyzed. The definition established by the authors of the primary publications will be acceptable if decided by consensus among the research team; it is consistent with the internationally accepted criteria. Results will not be combined when substantial heterogeneity (I ^2^>60%) is found.

The International Conference on Harmonization Guidelines defined SAE as any event that led to death, was life-threatening, caused hospitalization or prolongation of existing hospitalization, led to persistent or significant disability, or any congenital anomaly or birth defect ^14^. The National Cancer Institute Common Terminology Criteria for Adverse Events (CTCAE) will be followed for the grading of the adverse events ( https://ctep.cancer.gov/protocoldevelopment/electronic_applications/ctc.htm).

QoL analyzed by any general or specific validated scale will be acceptable.

### Methods for identification of studies

The literature search will be designed and launched through the Epistemonikos-L·OVE platform ( https://app.iloveevidence.com). This platform has been validated as a repository for the COVID-19 showing to be a highly comprehensive source of evidence ^15, 16^. The following approach will be used:

1. Identification of terms relevant to the population and intervention components of the search strategy, using Word2vec technology to the corpus of documents available in Epistemonikos Database.
2. Discussion of terms with content and methods experts to identify relevant, irrelevant and missing terms.
3. Creation of a sensitive boolean strategy encompassing all the relevant terms.

The main search source will be the Epistemonikos database ( https://www.epistemonikos.org), a comprehensive database of systematic reviews and other types of evidence, maintained by screening multiple information sources to identify systematic reviews and their included primary studies, including Cochrane Database of Systematic Reviews, MEDLINE, EMBASE, CINAHL, PsycINFO, LILACS, DARE, HTA Database, Campbell database, JBI Database of Systematic Reviews and Implementation Reports, EPPI-Centre Evidence Library ^16^.

An additional search will be performed on MEDLINE in order to identify randomized trials and non-randomized studies. The searches will cover the inception date of each database. No publication status or language restriction will be applied to the searches in Epistemonikos or to the additional searches. We will apply validated filters to identify clinical trials in the MEDLINE database.

### Boolean search strategy

***Epistemonikos***

(leukem* OR leukaem* OR leucem* OR leucaem* OR lymphoma* OR lymphobl* OR ((hemato* OR haemato* OR lymph* OR myelo*) AND (malignan* OR malignan*))) AND ((("car-engineered" OR "car-modified" OR "receptor-modified" OR chimeric* OR adoptive* OR redirected* OR engineered*) AND ("t cell" OR "t cells" OR "t-cell" OR "t-cells")) OR "car-t" OR "car t" OR "car-ts" OR "car ts" OR "car t-cell" OR "car t-cells")

***Medline. PUBMED***

(leukem* OR leukaem* OR leucem* OR leucaem* OR lymphoma* OR lymphobl* OR ((hemato* OR haemato* OR lymph* OR myelo*) AND (malignan* OR malignan*))) AND ((("car-engineered" OR "car-modified" OR "receptor-modified" OR chimeric* OR adoptive* OR redirected* OR engineered*) AND ("t cell" OR "t cells" OR "t-cell" OR "t-cells")) OR "car-t" OR "car t" OR "car-ts" OR "car ts" OR "car t-cell" OR "car t-cells")

Results from these searches will be automatically included in the L.OVE platform of the Epistemonikos Foundation ( https://iloveevidence.com/).

***Other sources***

We will carry out a manual search for reviewing the reference list of included studies, guidelines, narrative reviews and any other document of interest in an attempt to identify additional references that might be potentially eligible and to keep monitoring for the new evidence.

### Study selection

The L·OVE platform repository will automatically incorporate all the references that meet the criteria of the research question. The titles and abstracts of the identified references will be independently screened by at least two authors. Full texts of all potentially eligible references will be retrieved to make a final decision on their inclusion. Reasons for the exclusion of the identified references will be registered. The screening process will be conducted through Rayyan-Intelligent Systematic Review software whenever necessary ( https://www.rayyan.ai/). Results of the screening process will be shown in a PRISMA ^17^ flow diagram.

### Data extraction

A specific data extraction form will be designed to incorporate data obtained from the included studies. Information will be independently extracted by two authors, and cross-checked by another author. Discrepancies will be addressed by discussion, and if necessary a third author will be also involved.

The following information will be extracted: 1) general information of the studies, study design, number of participants, follow-up time, type of centre (Unicenter, multicenter), country, study sponsor and sources of funding; 2) Trial methods and conduction, such as randomization, blinding and follow-up time; 3) characteristics of the participants including age, sex, type of pathology, stage, number of previous lines, previous HSCT, the median time from diagnosis, percentage with high-risk cytogenetics, percentage with extramedullary disease; 4) Intervention characteristics such as T-cell origin (allogenic or autologous), transfection/transduction method (lentivirus, retrovirus, electroporation, or others), target antigen, CAR costimulatory domain, infusion dose; 5) Characteristics of the comparator; 6) Results of the measured outcomes.

The need for interleukin receptor antagonists to treat cytokine-release syndrome in the participants in the CAR-T arm will be analyzed.

We will contact the authors of the primary studies in case of missing information in the retrieved studies. Whenever possible, individual patient data and clinical study reports of included studies will be obtained by contacting corresponding authors, sponsors and promoters.

### Data analysis

***Risk of bias assessment***

In the case of RCTs, the risk of bias will be evaluated using the Cochrane Risk of Bias Tool. This tool considers the following domains: selection bias, performance bias, detection bias, attrition bias, reporting bias, and other bias ^18^. We will rate the overall risk of bias of each study as low, moderate, high or unclear risk of bias.

The risk of bias of non-randomized studies of intervention will be assessed using the ROBINS-I tool ^19^. The tool evaluates the following domains: bias due to confounding, selection of participants, classification of interventions, deviation from intended interventions, missing data, measurement of outcomes, and bias in selection of the reported result. According to these domains, we will judge the overall risk of bias as low, moderate, serious, critical or no information.

***Measures of treatment effect***

In the case of binary outcomes results will be presented as the proportion of patients who suffered the corresponding analyzed event. We will use hazard ratio (HR), risk ratio (RR) and Odds ratio (OR) for summarizing the results as appropriate. We will also estimate the risk difference in absolute terms and also the number needed to treat for an additional beneficial or harmful outcome when applicable. We will determine the 95% confidence intervals (CI).

For quantitative data, mean difference (MD) or standardized mean difference (SMD) will be estimated as appropriate. We will also determine the 95%CI.

***Data synthesis***

Results will be analyzed separately according to each specific study design. Data will be analyzed following an intention-to-treat approach. We will combine outcomes across trials using a fixed-effects model when applicable.

The heterogeneity of treatment effect among studies will be analyzed by estimating the I² statistic. We will consider I²>60% as indicative of substantial heterogeneity, whose possible reasons will be explored by carrying out subgroup analyses. We will carry out meta-analyses when data from more than one study are available and unless substantial heterogeneity (I²>60%) is found, whose results will be represented using forest plots. We will carry out meta-analyses using Review Manager Software (RevMan version 5.4).

Results will be presented in a narrative form in case there is one only study that provides data for an outcome variable or when data obtained from different studies cannot be combined.

***Subgroup analysis***

When possible, overall survival rate, overall response rate and total grade 3 or higher adverse events will be analyzed according to the following aspects:

-Type of CAR-T therapy (antigen type, costimulatory domain)

-Type of hematologic disease

-Type of comparator

-Age group (≥18 years and <18 years)

-Treatment line

-Tumor burden

-Cancer stage

***Sensitivity analysis***

Sensitivity analyses will be performed in terms of the following criteria:

-Analysis excluding studies with a high risk of bias.

-Analysis excluding industry-sponsored studies.

### Certainty of evidence

The quality of the evidence for each outcome variable will be analyzed using GRADE methodology, which includes the assessment of five main aspects: risk of bias, directness of the evidence, consistency among trials’ results, the precision of effect estimates, and risk of publication bias ^20^.

Two reviewers will independently assess the quality of the evidence. All possible discrepancies will be discussed until consensus and by the involvement of a third author, if necessary. Results of this assessment will be presented in a Summary of Findings table generated in the tool GRADEpro ® ( https://www.gradepro.org/)

### Evidence monitoring and surveillance plan

In order to maintain the living evidence process for this review, the Epistemonikos-L.OVE platform ( https://iloveevidence.com/) will be used as a technological enabler to support evidence identification, screening, and selection. We will keep a living search in the L·OVE platform to detect systematic reviews and randomized controlled trials. Additionally, every three months, we will manually search for ongoing studies in the WHO International Clinical Trials Registry Platform and in clinicaltrials.gov.

One reviewer will be in charge of assessing the evidence that has entered the specific question in the L.OVE platform every month and applying the selection criteria presented above. If a potentially eligible study is found, a second reviewer will confirm its eligibility by reading the full text. Results of evidence surveillance will be collected and kept as part of the study records. Information on the PRISMA figure will be updated accordingly. The PICO question and criteria for selecting studies will be revised and changed accordingly during the Living Evidence process every time new eligible studies are incorporated into the evidence synthesis on main outcomes or every four months, whichever is reached first. .

We will carry out data extraction for all new eligible studies. The data synthesis will be updated immediately after that, and the quality of the evidence will be evaluated following the GRADE approach accordingly, looking for changes on the certainty assessment results.

The living process for this question will end when the certainty of the evidence on the updated estimates for the desirable and undesirable effects becomes high or after 12 months of surveillance whatever is reached first.

### Statistical considerations for the living evidence synthesis

The inclusion of new studies identified as part of evidence surveillance and reporting on the outcomes of interest will follow this approach: We will perform an updated meta-analysis for each of the outcomes of interest including data obtained from the new studies. Data will be analyzed using a fixed-effect model. Heterogeneity among included studies will be analyzed by using the I ^2^ statistics. If new heterogeneity is detected (Compared to the previous meta-analysis, new heterogeneity appears or increases), we will explore its potential sources by reviewing the new studies against previously included studies in order to identify reasons that may explain inconsistent results among studies. In the presence of unexplained heterogeneity (I ^2^> 60%), data will not be meta-analyzed, and the results of the evidence synthesis will be explained narratively.

## Dissemination plan

We plan to communicate our review results (from baseline review and its up-dates) by publication in a scientific journal. We will elaborate technical reports to the healthcare providers, decision-makers, and clinical committees. We will share the results through our social media channels and in Open science Framework ( https://osf.io/). The base line report as well as the periodical updates will be available on the Living Evidence to Inform Health Decisions project website ( https://livingevidenceframework.com/en/).

If during the living process, new relevant results that imply changes in the current clinical practice are identified, we will update the report of this review and disseminate the new evidence and conclusions among potential users through all the above described communication channels.

## Study status

The searching and screening phases have been completed at the time of this submission.

## Data availability

### Underlying data

No data are associated with this article.

### Extended data

Open Science Framework: LIVING EVIDENCE TO INFORM HEALTH DECISIONS. 10.17605/OSF.IO/V6HDX

### Reporting guidelines

Open Science Framework: PRISMA-P checklist for “Efficacy and safety of Chimeric Antigen Receptor T-Cell (CAR-T) therapy in hematologic malignancies: a living systematic review (Protocol)”. 10.17605/OSF.IO/V6HDX

Data are available under the terms of the Creative Commons Zero “No rights reserved” data waiver (CC0 1.0 Public domain dedication).
