# Supplementary material for: Efficacy and safety of chimeric antigen receptor T-cell (CAR-T) therapy in hematologic malignancies: a living systematic review (protocol)
Source: Open Res Eur. 2024 May 21;2:38. Originally published 2022 Mar 15. [Version 2] doi: 10.12688/openreseurope.14390.2 (PMC11140298; doi:10.12688/openreseurope.14390.2)
Supplement: Peer review history

# Reviewer report: Reviewer response for version 2

**DOI:** 10.21956/openreseurope.18861.r40753

**Published:** 2024-05-30

**Authors:** Lee M Greenberger

**Affiliations:** 1 The Leukemia and Lymphoma Society, Rye Brook, NY, USA

All the edits are acceptable and ths work should be accepted.

Is the study design appropriate for the research question?

Yes

Is the rationale for, and objectives of, the study clearly described?

Yes

Are sufficient details of the methods provided to allow replication by others?

Yes

Are the datasets clearly presented in a useable and accessible format?

No

Reviewer Expertise:

Grant and venture philanthropy funding for blood cancer.

I confirm that I have read this submission and believe that I have an appropriate level of expertise to confirm that it is of an acceptable scientific standard.

# Reviewer report: Reviewer response for version 1

**DOI:** 10.21956/openreseurope.15530.r36013

**Published:** 2023-12-04

**Authors:** Le Wang

**Affiliations:** 1 Jiangsu University, Zhenjiang, Jiangsu, China

Strengths:

- Clear Objectives: The paper sets a defined goal to determine the efficacy and safety of CAR-T therapy in hematological malignancies and compare it with other current therapies.
- Sound Methodology: The use of an active systematic review approach adheres to the PRISMA-P guidelines.
- Comprehensive Study Design: Inclusion of both randomized controlled trials and non-randomized original studies offers a well-rounded research design.
- Detailed Data Extraction and Analysis: Utilization of the Cochrane risk of bias tool and ROBINS-I for bias risk assessment, and fixed effect model analysis for results, is commendable.

Suggestions for Improvement:

- Data and Timeframe Scope: Given the rapid advancements in the CAR-T field, the 12-month monitoring period might be insufficient to reflect the latest research and long-term outcomes.
- Quality of Life Measurements: The methodology and integration of quality of life (QoL) results need clearer delineation.
- Real-world Data Application: Incorporation of real-world data could enhance the breadth and applicability of the analysis.
- Economic Considerations: Given the high cost of CAR-T therapy, analyzing its economic impact is crucial for clinical decision-making.
- Completeness of Reporting: Enhance the detailing of ongoing and completed trials for a better understanding of the study's context and setting.

Conclusion and Recommendation:

 This paper presents a promising study aimed at comprehensively evaluating the efficacy and safety of CAR-T therapy in hematological malignancies. However, it is recommended that the authors consider extending the monitoring period, including analyses of economic and real-world data, and clarifying the methodology for comparing QoL data. These improvements will enhance the practical applicability and impact of the research.

 Recommendation for indexing: Major Revision Required

 Rationale: The study is valuable but requires significant enhancements in terms of data scope, economic analysis, and methodological clarity to be suitable for publication. After addressing these major concerns, the paper could provide a more impactful and comprehensive insight into the CAR-T therapy landscape in hematological malignancies.

Is the study design appropriate for the research question?

Yes

Is the rationale for, and objectives of, the study clearly described?

Yes

Are sufficient details of the methods provided to allow replication by others?

Yes

Are the datasets clearly presented in a useable and accessible format?

Yes

Reviewer Expertise:

CAR-T, breast cancer

I confirm that I have read this submission and believe that I have an appropriate level of expertise to confirm that it is of an acceptable scientific standard, however I have significant reservations, as outlined above.

# Response

**Published:** 2024-04-26

**Authors:** Maria Ximena Rojas Reyes

**Affiliations:** INSTITUT DE RECERCA DE L'HOSPITAL DE LA SANTA CREU I SANT PAU FUNDACION - IR-HSCSP, Spain

Strengths:

- Clear Objectives: The paper sets a defined goal to determine the efficacy and safety of CAR-T therapy in hematological malignancies and compare it with other current therapies.
- Sound Methodology: The use of an active systematic review approach adheres to the PRISMA-P guidelines.
- Comprehensive Study Design: Inclusion of both randomized controlled trials and non-randomized original studies offers a well-rounded research design.
- Detailed Data Extraction and Analysis: Utilization of the Cochrane risk of bias tool and ROBINS-I for bias risk assessment, and fixed effect model analysis for results, is commendable.

Suggestions for Improvement:

- Data and Timeframe Scope: Given the rapid advancements in the CAR-T field, the 12-month monitoring period might be insufficient to reflect the latest research and long-term outcomes.

Thank you for the comment. We have broadened the initial follow-up to set a 36-month monitoring period based on our existent resources. In any case, we agree CAR-T field is rapidly evolving and a longer monitoring duration would be ideal. In this regard, our team is committed to extend the monitoring process as long as new high quality evidence arises and our resources allows for it. 

- Quality of Life Measurements: The methodology and integration of quality of life (QoL) results need clearer delineation.

QoL constitutes in our project a secondary safety outcome. We will accept any general or specific validated scale used to estimate QoL. Results that refer to specific domains of a QoL scale will be analyzed separately from results that refer to the overall results of the scale. In the event the identified studies reported QoL using the same scale, these data will be meta-analyzed and reported as mean difference (MD) with 95% confidence interval (95%CI). On the contrary, if the studies included in the review were to use different validated scales for reporting QoL, data will be standardized before an aggregated standardized mean difference (SMD) with 95%CI is calculated.

- Real-world Data Application: Incorporation of real-world data could enhance the breadth and applicability of the analysis

Thank you for this appreciation. As stated in the current version of the protocol, randomized controlled trials (RCTs) have been prioritized given their higher quality of evidence, but observational studies with comparative designs have also been taken into consideration, specifically in cases where the evidence from RCTs is not sufficient. We agree that real-world data are also of great interest in this field, particularly when safety issues are assessed, and are not excluded in our inclusion criteria. Nonetheless, our experience with the original version of this review has showed us the great complexity of mixing experimental and observational designs in just one paper. Consequently, we highly appreciate your suggestion, which could probably be considered and addressed in separate publications.  

- Economic Considerations: Given the high cost of CAR-T therapy, analyzing its economic impact is crucial for clinical decision-making.

Thank you. This living systematic review has been designed to focus on CAR-T efficacy and safety. We agree on the importance of economic considerations, but these are out of the scope in the current project, as they may depend on the setting, context and time point in which CAR-T therapy is applied. Therefore, results from studies conducted at distant moments in time, and in diverse countries and settings cannot be directly extrapolated to other contexts, since unit costs can be highly variable.

- Completeness of Reporting: Enhance the detailing of ongoing and completed trials for a better understanding of the study's context and setting.

Thank you for this valuable comment. Following your suggestion, the Background section has been broadened including two recent systematic reviews and several published and ongoing clinical trials on the topic. In our view, both of them help understand the context of past, present and future CAR-T evidence. In this sense, the following text has been added: *“A recently published systematic review focused on randomized controlled trials (RCTs) analyzing the efficacy and safety of CAR-T therapies versus high-dose salvage chemotherapy followed by autologous stem cell transplantation as second-line treatment of relapsed or refractory large B-cell lymphoma (13). However, this review shows several limitations, such as the lack of diverse comparators and hematologic diseases, and methodological concerns with the pooled analysis strategy. A second systematic review showing remarkable differences with our living project was also identified, providing mixed results in adults and children, an updated search 6 months shorter than our study, less detail on pre-specified exploratory outcomes and no data available on complementary observational evidence (14).”* *“Although more than a thousand CAR-T trials are being conducted* *(15)* *, those with the highest quality, randomized clinical trials are still scarce. Some data from RCTs in different hematological pathologies have already been published (BELINDA (16), ZUMA-7 (17), TRANSFORM (18), KarMMa-3 (19) or CARTITUDE-4 (20)), and other studies are currently underway (KarMMa-4, CARTITUDE-5, CARTITUDE-6 or DALY 2-EU).”* Conclusion and Recommendation:

 This paper presents a promising study aimed at comprehensively evaluating the efficacy and safety of CAR-T therapy in hematological malignancies. However, it is recommended that the authors consider extending the monitoring period, including analyses of economic and real-world data, and clarifying the methodology for comparing QoL data. These improvements will enhance the practical applicability and impact of the research.

 Recommendation for indexing: Major Revision Required 

 Rationale: The study is valuable but requires significant enhancements in terms of data scope, economic analysis, and methodological clarity to be suitable for publication. After addressing these major concerns, the paper could provide a more impactful and comprehensive insight into the CAR-T therapy landscape in hematological malignancies. **Is the rationale for, and objectives of, the study clearly described?** Yes **Is the study design appropriate for the research question?** Yes **Are sufficient details of the methods provided to allow replication by others?** Yes **Are the datasets clearly presented in a useable and accessible format?** Yes 

# Reviewer report: Reviewer response for version 1

**DOI:** 10.21956/openreseurope.15530.r35699

**Published:** 2023-11-22

**Authors:** LaQuisa Hill

**Affiliations:** 1 Department of Medicine, Baylor College of Medicine, Houston, TX, USA

The authors describe a protocol to develop a "living" systematic review of published CAR-T studies for hematologic malignancies.

 The rationale for the study is clearly described. However, it may be difficult to meet the objectives of efficacy and safety as many of the currently available studies are non-randomized single center or single arm multii-center studies (which will be excluded per methods). Also the timeframe of ongoing review for only 12 months will likely be insufficient to establish rigorous review and updated findings given the number of ongoing studies, and many new ones in development. It typically takes several years for randomized study data to be available so 12 months will likely be too short. 

 Other endpoints to consider are time from diagnosis to CAR-T treatment, time to response and duration of responses.

 While QoL outcomes are becoming increasingly important and utilized, it is not clear from the protocol how different methods for QoL will be compared and/or grouped to determine true impact.

 Also will there be separate reports for individual diseases? CAR-T products?

 No datasets were provided so unable to assess usability or accessibility.

Is the study design appropriate for the research question?

Yes

Is the rationale for, and objectives of, the study clearly described?

Yes

Are sufficient details of the methods provided to allow replication by others?

Yes

Are the datasets clearly presented in a useable and accessible format?

Not applicable

Reviewer Expertise:

Stem cell transplantation and cellular therapy

I confirm that I have read this submission and believe that I have an appropriate level of expertise to confirm that it is of an acceptable scientific standard, however I have significant reservations, as outlined above.

# Response

**Published:** 2024-04-26

**Authors:** Maria Ximena Rojas Reyes

**Affiliations:** INSTITUT DE RECERCA DE L'HOSPITAL DE LA SANTA CREU I SANT PAU FUNDACION - IR-HSCSP, Spain

The authors describe a protocol to develop a "living" systematic review of published CAR-T studies for hematologic malignancies. The rationale for the study is clearly described. However, it may be difficult to meet the objectives of efficacy and safety as many of the currently available studies are non-randomized single center or single arm multii-center studies (which will be excluded per methods). Also the timeframe of ongoing review for only 12 months will likely be insufficient to establish rigorous review and updated findings given the number of ongoing studies, and many new ones in development. It typically takes several years for randomized study data to be available so 12 months will likely be too short.  Thank you for the comments. As stated in the protocol, randomized controlled trials (RCTs) have been prioritized given their higher quality of evidence, but non-randomized and observational studies with comparative designs have also been taken into consideration, specifically in cases where the evidence from RCTs is not sufficient. Nonetheless, we have experienced high complexities to combine results from different study designs within a single publication. Thus, we are planning to focus on RCTs in subsequent versions of our living systematic review. We have broadened the initial follow-up to set a 36-month monitoring period based on our existent resources. In any case, we agree CAR-T field is rapidly evolving and a longer monitoring duration would be ideal. In this regard, our team is committed to extend the monitoring process as long as new high quality evidence arises and our resources allows for it.  

 Other endpoints to consider are time from diagnosis to CAR-T treatment, time to response and duration of responses. Thank you for the suggestion. We agree on including “time to response” and “duration of response” as additional secondary outcomes. 

 While QoL outcomes are becoming increasingly important and utilized, it is not clear from the protocol how different methods for QoL will be compared and/or grouped to determine true impact. Thank you for the comment. We have expanded the information in the protocol to clarify this point: “ *We will accept any general or specific validated scale used to estimate QoL. Results that refer to specific domains of a QoL scale will be analyzed separately from results that refer to the overall results of the scale. In the event the identified studies reported QoL using the same scale, these data will be meta-analyzed and reported as MD with 95%CI. On the contrary, if the studies included in the review were to use different validated scales for reporting QoL, data will be standardized before an aggregated SMD with 95%CI is calculated.*”   Also will there be separate reports for individual diseases? CAR-T products? Thank you for the comment. We have clarified in the protocol that we will perform separate analysis for individual diseases: “ *Results will be analyzed separately according to each specific study design and* *type of hematologic disease*”. As stated in the protocol, subgroup analyses of the main outcomes (overall survival rate, overall response rate and total grade 3 or higher adverse events) will be conducted according to the type of CAR-T product, among other factors. 

 No datasets were provided so unable to assess usability or accessibility. **Is the rationale for, and objectives of, the study clearly described?** Yes **Is the study design appropriate for the research question?** Yes **Are sufficient details of the methods provided to allow replication by others?** Yes **Are the datasets clearly presented in a useable and accessible format?** Not applicable 

# Reviewer report: Reviewer response for version 1

**DOI:** 10.21956/openreseurope.15530.r33594

**Published:** 2023-07-17

**Authors:** Lee M Greenberger

**Affiliations:** 1 The Leukemia and Lymphoma Society, Rye Brook, NY, USA

This protocol describes a systematic review of CAR T therapy for hematologic malignancies. The work will rely on previous CAR T studies, most of which have been conducted without randomized control arms. So, while the output of this protocol is needed and will assess performance of CAR Ts, trials, comparisons between CAR T results from independent trials are difficult. Ultimately, the analysis will be replaced by phase 3 comparison trials (e.g. vs SOC), which are just beginning to be conducted for early stage disease treatment.

 The endpoints for analysis are comprehensive, although time to response was not listed as an analysis endpoint.

 The monitoring process is only for 12 months.  As the CAR T field is rapidly changing, is this a long enough period to conduct the study? What is the justification for only 12 months?

 Will there be any effort to assess the financial toxicity of CAR T therapy as well as access and response in underserved populations?

 Are the authors considering data generated in real-world experience? There are multiple reports of real-world CAR T data including CD19 CAR T for ALL and CD19 CAR T for lymphoma.

 What quality of life instruments will be used and how do the authors intend to compare QoL if the measurements are done with different methods?

 It is not possible to evaluate the datasets, as no datasets were presented.

Is the study design appropriate for the research question?

Yes

Is the rationale for, and objectives of, the study clearly described?

Yes

Are sufficient details of the methods provided to allow replication by others?

Yes

Are the datasets clearly presented in a useable and accessible format?

No

Reviewer Expertise:

Grant and venture philanthropy funding for blood cancer.

I confirm that I have read this submission and believe that I have an appropriate level of expertise to confirm that it is of an acceptable scientific standard, however I have significant reservations, as outlined above.

# Response

**Published:** 2024-04-26

**Authors:** Maria Ximena Rojas Reyes

**Affiliations:** INSTITUT DE RECERCA DE L'HOSPITAL DE LA SANTA CREU I SANT PAU FUNDACION - IR-HSCSP, Spain

This protocol describes a systematic review of CAR T therapy for hematologic malignancies. The work will rely on previous CAR T studies, most of which have been conducted without randomized control arms. So, while the output of this protocol is needed and will assess performance of CAR Ts, trials, comparisons between CAR T results from independent trials are difficult. Ultimately, the analysis will be replaced by phase 3 comparison trials (e.g. vs SOC), which are just beginning to be conducted for early stage disease treatment.

 The endpoints for analysis are comprehensive, although time to response was not listed as an analysis endpoint. Thank you for this suggestion. We agree on including “time to response” as an additional secondary outcome.   The monitoring process is only for 12 months.  As the CAR T field is rapidly changing, is this a long enough period to conduct the study? What is the justification for only 12 months? Thank you for this question. We have broadened the initial follow-up to set a 36-month monitoring period, based on our existent resources. In any case, we agree CAR-T field is rapidly evolving and a longer monitoring duration would be ideal. In this regard, our team is committed to extend the monitoring process as long as new high quality evidence arises and our resources allows for it. 

 Will there be any effort to assess the financial toxicity of CAR T therapy as well as access and response in underserved populations? Thank you. This living systematic review has been designed to focus on CAR-T efficacy and safety. We agree on the importance of economic considerations but these are out of the scope in the current project, as they may depend on the setting and context in which CAR-T therapy is applied.

 Are the authors considering data generated in real-world experience? There are multiple reports of real-world CAR T data including CD19 CAR T for ALL and CD19 CAR T for lymphoma. Thank you for this question. As stated in the current version of the protocol, randomized controlled trials (RCTs) have been prioritized given their higher quality of evidence, but observational studies with comparative designs have also been taken into consideration, specifically in cases where the evidence from RCTs is not sufficient. Nonetheless, we have experienced high complexities to combine results from different study designs within a single publication. Thus, we are planning focus on RCTs in subsequent versions of our living systematic review. We agree that real-world data are also of great interest in this field and are not excluded in our inclusion criteria but, in our view, it should be addressed in a separate publication.   

 What quality of life instruments will be used and how do the authors intend to compare QoL if the measurements are done with different methods? Thank you for this interesting comment. We will include the following statement in the “Data analysis” section of the protocol: “ *We will accept any general or specific validated scale used to estimate QoL. Results that refer to specific domains of a QoL scale will be analyzed separately from results that refer to the overall results of the scale. In the event the identified studies reported QoL using the same scale, these data will be meta-analyzed and reported as MD with 95%CI. On the contrary, if the studies included in the review were to use different validated scales for reporting QoL, data will be standardized before an aggregated SMD with 95%CI is calculated*.”

**It is not possible to evaluate the datasets, as no datasets were presented.** Thank you. As it is a protocol, there are no datasets associated with it. **Is the rationale for, and objectives of, the study clearly described?** Yes **Is the study design appropriate for the research question?** Yes **Are sufficient details of the methods provided to allow replication by others?** Yes **Are the datasets clearly presented in a useable and accessible format?** No
